# The factor structure and psychometric properties of the Chinese version of the Positive and Negative Suicide Ideation Inventory (PANSI-C) in a non-clinical sample of Chinese adolescents

**DOI:** 10.1186/s12991-021-00366-y

**Published:** 2021-09-28

**Authors:** Wei Chen, Tao Yang, Rongfen Gao, Guyin Zhang

**Affiliations:** 1 School of Psychology, Guizhou Normal University, Huaxi University Town, GuiAn District, Guiyang, 550001 China; 2 Center for Big Data Research in Psychology, Guizhou Normal University, Guiyang, China

**Keywords:** Suicide ideation, Positive and Negative Suicide Ideation (PANSI), Psychometric properties, Measurement invariance

## Abstract

**Backgrounds:**

The Positive and Negative Suicide Ideation (PANSI) Inventory is a widely used self-report questionnaire which is designed to comprehensively evaluate the protective factors and negative risk factors associated with suicidal behaviors among adolescents. The present study aimed to evaluate the psychometric properties and measurement invariance of the Chinese version of the PANSI in a non-clinical sample of Chinese adolescents.

**Methods:**

Participants (*N* = 1198) were Chinese middle school students aged 11–17 years (44.8% boys and 51.9% girls, 3.3% missing values) in Guizhou Province. All participants completed the Chinese version of the Positive and Negative Suicide Ideation Inventory (PANSI-C), the Rosenberg self-esteem scale (RSE), and the suicide probability scale (SPS). Cronbach’s alpha coefficients, confirmatory factor analysis, Pearson’s correlations, and multigroup confirmatory factor analysis tests were conducted thereafter.

**Results:**

The results showed that the Cronbach’s alpha coefficients for the two subscales of the PANSI-positive suicide ideation and the PANSI-negative suicide ideation were .696 and .915, respectively. The confirmatory factor analysis supported the fit of the two-factor model as the best fitting model [Chi-square goodness of fit = 703.859, *p* < .001, degrees of freedom = 76, comparative fit index = .919, Tucker–Lewis index = .903, standardized root mean square residual = .047, root mean square error of approximation (90% CI) = .083 (.077, .089)]. Positive suicide ideation had negative correlations with the SPS and positive correlations with the RSE, whereas the negative suicide ideation had positive correlations with the SPS and negative correlations with the RSE. All correlations were statistically significant (*p* < .001), demonstrating the criterion validity of the PANSI-C. Moreover, the strict measurement invariance of the PANSI-C was supported across gender, single-parent and non-single-parent households groups, and the strong measurement invariance was supported across age.

**Limitations:**

The feasibility of this study is limited to Chinese normal adolescents and lack of clinical samples.

**Conclusion:**

Empirical support for the reliability and validity of the PANSI-C was found. The PANSI-C instrument is found to be useful in assessing positive and negative suicide ideation in Chinese normal adolescents.

## Introduction

Suicide is a widespread issue of concern worldwide. According to the World Health Organization [54], suicide was the 18th leading factor of death in the world in 2016, and the second leading factor of death among people aged 15–29. Approximately 800,000 people died of suicide in 2016, with 1 person dying by suicide every 40 s, and even more attempting suicide [54]. According to the survey of WHO, mortality is expected to increase to one person every 20 s [54], the issue of suicide has become more serious. Suicide is of increasing concern worldwide, due to its heightened impact on adolescents, as well as the serious impacts it can have on other individuals, families, and societies related to the individual attempting suicide. As previous studies have shown, the incidence of suicide ideation among adolescents was 10.72–12.1% [24], with suicidal intention and planning at 8.1% [32]. In China, more than 10,000 teenagers died by suicide each year [36]. As such, there have been numerous efforts made to improve abilities to identify adolescents at elevated risk of suicide, so as to prevent and effectively reduce the youth suicide rate [15, 37].

Suicidal behavior is a series of complex processes, from suicidal ideation, suicide plan, suicidal attempt, and suicide death [19]. Suicidal ideation is the main risk factor for suicidal behavior, refers to the idea that the individual wants to end life [4]. Although individuals with suicidal ideation do not necessarily died by suicide [31], suicidal ideation is indeed an important predictor of suicide risk [18, 22]. Studies have shown that the peak of suicidal ideation occurs in adolescence, with the incidence rising from less than 1% at the age of 10 to 17% at the age of 18 [32]. In adolescence, adolescents have the cognitive ability to think and evaluate death, but they are in an immature state in terms of cognitive control and emotional response. This imbalance can easily lead to suicidal ideation [28]. Therefore, paying attention to the related factors of suicidal ideation among adolescents will not only help screen out high-risk groups, but also help maintain the mental health of adolescents [26].

The development of effective measurement instruments can be an important means for identifying and studying suicidal ideation. A number of instruments have been developed to assess adolescents’ suicide ideation. The Beck scale for suicide ideation (BSI) [3], which is used to evaluate the status of one’s suicidal ideation over the past week, has 19 items and results in scores in two dimensions, one is suicide ideation and the other is suicidal tendency. The higher the score, the higher one’s risk of suicidal ideation and suicide is. The suicidal ideation questionnaire (SIQ) [40], consists of 30 items that evaluate specific thoughts and cognitions about suicide and death over the past month. The higher the SIQ score, the more serious one’s suicidal ideation is. The modified scale for suicide ideation (MSSI) [29] includes 18 items; the higher the score, the more serious one’s suicidal ideation is. Another scale, such as the suicidal ideation scale (SIS) [42], has been designed for college students, and uses 10 items to measure suicidal ideation over the past year. Although each of these measurements has merits, their limitations cannot be ignored. Most people believe that suicidal behavior is closely related to risk factors, such as psychological distress or psychiatric disorders [17], and research on suicidal behavior has also focused on the risk factors of suicidal ideation, and rarely considers the protective factors.

It has been noticed, however, the factors affecting suicidal ideation or behavior may be multidimensional [39]. For example, individuals with suicidal thoughts may also express their desire to survive [48]. The Positive and Negative Suicide Ideation (PANSI) Inventory combined with risk and protective factors (i.e., positive ideation and negative suicide ideation) to evaluate individual suicidal ideation [35]. In the original study, the PANSI showed good reliability and a two-factor structure among college students [35]. The study tested the reliability and validity of the PANSI with diverse samples of young adults, high school students, and psychiatric inpatients, and the results showed good reliability and a stable two-factor structure [30, 33, 34]. A large number of studies have verified the measurement performance of the scale. For instance, the PANSI has been shown to be an effective and reliable instrument to measure the severity of suicidal ideation among clinical outpatients in Malaysia [43]. The PANSI has also shown good psychometric properties among Korean middle school students [23], as well as good reliability and validity among Nigerian college students and Colombian students [1, 49]. In China, research involving a sample of middle school students and senior high school students in Taiwan found that Cronbach’s alpha coefficients of the PANSI-negative suicide ideation (PANSI-NSI) was .94, and that the PANSI-positive ideation (PANSI-PI) was .86. The two-factor model has also been replicated. However, participants in this study were limited to urban areas [6]. Another study also found that the PANSI had good reliability and validity among high school students in Henan, China [50].

Measurement invariance means that, given a latent factor, the conditional distribution of the observed variables is invariant across groups, namely there is no measurement bias associated with a specific group in different conditions [7]. Studies have shown that when the same measurement scale is applied in different situations, the measurement characteristics are likely to change [10]. Different groups (e.g., country, gender, and age) may have different understandings of the items in a particular scale [14, 44, 46]. Although some studies have compared the differences in PANSI scores between genders, the conclusions have been inconsistent. In the PANSI-NSI subscale, males have generally scored significantly higher than females, but there has been no significant difference between the genders in the PANSI-PI subscale [1]. Some studies have shown that girls’ scores in the PANSI-NSI are significantly higher than those of boys [6], while others have shown that there is no significant difference between in PANSI scores between males and females [30, 33, 35]. Single-parent family children refer to children under the age of 18 who are raised by their father or mother alone due to the divorce of their parents or the death of one party, or other reasons, and who do not have the ability to live independently [5]. Defects in family structure and absence of parent education make children from single-parent families face huge challenges in psychological development and social adaptation [16, 51], specifically showing more problems in self-esteem, social anxiety, anti-social behavior [8, 38, 47]. If researchers want to use the PANSI scale to explore the actual differences in suicidal ideation between different groups, it is necessary to ensure that the scale has the invariance of cross-group measurement [13]. Furthermore, we tested whether the PANSI remained unchanged in the youth age category. In the World Health Organization [53] age classification, the age of adolescents is 10–19 years. In addition, adolescents are divided into younger adolescents (10–14 years) and older adolescents (15–19 years). Therefore, we divided the age group into 11–14 years and 15–17 years to measure the invariance in the current study. In summary, this study examined the measurement invariance of gender, single-parent and non-single-parent households and age of the PANSI.

Based on the above review of existing literature, this study aimed to evaluate the reliability and validity of the PANSI and its measurement invariance on variables such as gender, single-parent and non-single-parent household and age so as to provide scientific basis for further research in related fields.

## Methods

### Translation procedure

We first used the method of “translation and back-translation”, with separate translations performed by three graduate students in psychology which were then compared and used to form the first draft of the measurement. Next, a group consisting of a psychology professor and eight postgraduates discussed and revised the first draft to form the second draft of the PANSI. After that, a senior professor of psychology was invited to join the discuss and revise the measure so that the language was accurate, fluent, and in line with Chinese expression habits so as to achieve equivalence between the Chinese and English versions, producing a final, formal questionnaire that formed the Chinese version of the PANSI (PANSI-C), see Table 1.

**Table 1:** The Chinese translation of the PANSI (PANSI-C)

PANSI-NSI
1.因为达不到别人的要求而考虑过自杀。
3.对未来感到绝望, 有轻生念头。
4.感觉和某人的关系很不好, 想自杀。
5.无法完成人生中重要的事情而想过自杀。
7. 个人问题无法解决时, 想自杀。
9.觉得自己很失败而有轻生的念头。
10.在面对不能解决的问题时, 除了自杀别无选择。
11.感到很孤独和悲伤, 为了结束痛苦而想自杀。
PANSI-PI:
2.我觉得自己能掌控生活中的大多数情况。
6.对生活很满意, 觉得未来充满希望。
8.学习上很顺利, 我感到很愉快。
12.我有信心处理好生活中的大部分问题。
13.感觉生命是值得活下去的。
14.我对未来充满信心。

### Subjects and data collection

In the convenient sampling of the four middle school classes, students read the informed consent form, and those who agree to participate will get small gifts after completing the questionnaire. The committee of the School of Psychology of Guizhou Normal University approved the study. Both the students and their parents had been informed that participation was voluntary, that results would be reported only in aggregate, and that the study responses and data management would be kept confidential. Use the following inclusion criteria: (a) no psychiatric disorders, (b) suicidal ideation occurred in the past 2 weeks, and (c) fully completing the survey. Study participants were comprised of 1198 middle school students with an average age of 13.86 (SD = 1.60), where 44.8% were boys, 51.9% were girls, and 3.3% were missing this data. Regarding where they lived, 40.8% lived in rural areas, 53.2% in urban areas, and 6% were missing this data. Most of the participants had siblings (*n* = 914, 76.3%), and the vast majority of them came from non-single-parent households (*n* = 1021, 85.2%). See Table 2 for further details.

**Table 2:** Frequency distribution of adolescents’ demographical characteristics (*N* = 1198)

Variables	Groups	*N*	%
Gender	Boy	537	44.8
	Girl	621	51.9
	Missing value	40	3.3
Age	11	1	.1
	12	65	5.4
	13	333	27.8
	14	406	33.9
	15	259	21.6
	16	75	6.3
	17	7	.6
	Missing value	52	4.4
Address	Rural	489	40.8
	Urban	637	53.2
	Missing value	72	6
Grade	First grade	424	35.4
	Second grade	420	35.1
	Third grade	315	26.3
	Missing value	39	3.3
Only child	Yes	237	19.8
	No	914	76.3
	Missing value	47	3.9
Single-parental family	Yes	124	10.4
	No	1021	85.2
	Missing value	53	4.4
Want a younger sibling	Yes	399	33.3
	No	705	58.8
	Missing value	99	7.9

### Measurements

#### Demographic information

Demographic information included participants’ gender (i.e., boy or girl), age, home address (i.e., rural or urban), grade (i.e., first, second, or third grade), whether they were an only child (i.e., yes/no), whether they lived in a single-parent family (i.e., yes/no), and whether they wanted to have a sibling (i.e., yes/no).

#### Positive and Negative Suicide Ideation Inventory (PANSI)

Suicidal ideation was measured by the Positive and Negative Suicide Ideation (PANSI) Inventory [35]. The PANSI evaluates both the protective and risk factors associated with suicidal ideation, and comprises two dimensions (14 items total): positive ideation (PANSI-PI,6 items) and negative suicide ideation (PANSI-NSI; 8 items). PANSI-NSI and PANSI-PI examined the frequency of specific negative thoughts (e.g., failure to accomplish something important) or positive thoughts (e.g., excited about doing well at school or work) related to suicidal behavior [35]. Participants used a Likert scale ranging from 1 (i.e., “none of the time”) to 5 (i.e., “most of the time”) to assess the frequency they experience suicidal ideation. Higher scores indicate more positive or negative suicide ideation, depending on the item’s particular subscale. The Cronbach’s *α* coefficients of the PANSI-NSI and PANSI-PI in this study were .915 and .696, respectively.

#### Rosenberg self-esteem scale (RSE)

Based on previous studies on suicidal ideation, the Rosenberg self-esteem scale (RSE) scale was selected as criterion instruments [23, 43]. The Rosenberg self-esteem scale (RSE) is a 10-item self-report questionnaire that assesses individual self-esteem [41]. The SES we used is a Chinese version translated by Yang and Wang [55]. Participants use a Likert scale ranging from 1 to 4. The higher the score, the higher one’s level of self-esteem is. The Cronbach’s alpha coefficient of the SES in this study was .826.

#### Suicide probability scale (SPS)

According to the research of Osman et al. [35], the SPS was selected as the effective standard instrument. The suicide probability scale (SPS) was developed by Cull and Gill [11], it includes four subscales: Hopelessness, Negative Self-Evaluation, Hostility and Suicide Ideation. The Chinese version of the SPS was translated by Liang and Yang [25]. This study uses the suicide ideation subscale, including 8 items, to measure the frequency of suicidal ideation in the past week. Participants use a Likert scale ranging from 1 to 4, with higher scores indicate the risk of suicide. The Cronbach’s alpha coefficient of the SPS in the present study was .799.

### Statistical analysis

Statistical Packages for Social Sciences (SPSS) version 25.0 and Mplus version 8.3 were used to perform the statistical analyses.

SPSS 25.0 was used to make the most basic descriptive statistics. Cronbach’s alpha coefficients were used to evaluate the reliability of the PANSI-C. A value of Cronbach’s alpha coefficients < .60 was considered to be insufficient; .60–.69 was marginal; .70–.79 was considered acceptable; .80–.89 was considered good; ≥ .90 was considered excellent [2]. Some researchers have pointed out, based on experience, that when the coefficients of skewness and kurtosis (absolute values) are less than 2 and 7, respectively, the Maximum Likelihood (ML) estimation method is acceptable [12, 52]. The absolute value of skewness of the data in the current study ranged from .007 to 1.899, and the absolute value of kurtosis was between .046 and 2.996. It can be seen ML can also be used to obtain reasonable parameter estimation results. We used confirmatory factor analysis to fit the model, and we used the approximate root mean square error of approximation (RMSEA) and its 90% CI, the comparative fit index (CFI), the Tucker–Lewis index (TLI), the standardized root means square residual (SRMR), and other fitting indicators to evaluate the degree of model fit. Past research supports that, if the CFI or TLI are more than .90 and the SRMR is less than .08, then RMSEA rates approximating .06 or lower indicate a good fit, .07–.08 an acceptable fit, .08–.10 a limited fit, and > .10 as unfit [21, 45]. The Pearson correlation coefficient was used to evaluate the correlation between the PANSI-C and each scale. Correlation intensity has been explained by Colton [9] as follows: 0–.25 = irrelevant or very small correlation, .26–.50 = general correlation, .51–.75 = moderate correlation, and .76–1.00 = complete correlation. Correlations were used to analyze the construct validity of the PANSI-C. The test for measurement invariance was a comparison of a series of nested models. Since the Chi-square test is extremely sensitive to the sample size, the larger the sample size, the more significant the result of the Chi-square test. As the sample size continues to increase, even small changes will cause significant differences [20]. Based on the above considerations, this study uses the difference in model fitting index between groups (∆CFI, ∆TLI) and ∆RMSEA as a reference index for measuring invariance. If configural invariance is obtained, then it means that the composition of latent variables is the same among different groups. If weak invariance is established, indicating that the factor loadings between the groups are equal. Strong invariance is used to test whether the intercepts of the observed variables are equal. The strict invariance model is used to test whether the error variances between different groups are equal [27]. Some researchers pointed out that when using the ∆CFI, ∆TLI and ∆RMSEA values to compare nested models, the measurement invariance model is acceptable when ∆CFI(TLI) ≤ .01 and ∆RMSEA ≤ .015 [7].

## Results

### Reliability

In this study, for the PANSI-C subscales, the Cronbach’s alpha coefficients were .696 and .915, respectively, showing acceptable reliability for both. The Cronbach’s alpha coefficients of the PANSI-C subscales and the other scales are shown in Table 3.

**Table 3:** Correlations of PANSI-C with other subscales

Variable	1	2	3	4	*M*(*SD*)	Alpha
1.PANSI-PI	1				20.82 (4.45)	.696
2.PANSI-NSI	− .476***	1			13.31 (6.89)	.915
3.RSE	.567***	− .509***	1		28.22 (5.50)	.826
4.SPS	− .409***	.707***	− .532***	1	13.64 (4.52)	.799

*PANSI-PI* the positive and negative suicide ideation-positive ideation, *PANSI-NSI* the positive and negative suicide ideation-negative ideation, *RSE* the Rosenberg self-esteem scale, *SPS* the suicide probability scale

** *p* < .01, *** *p* < .001

### Validity

#### Construct validity

Since the PANSI scale has shown a stable two-factor structure in previous studies, the current study directly verified the PANSI-C’s two-factor structure. The confirmatory factor analysis results showed acceptable fitting indices of the two-factor model—*χ*^2^ = 703.859, *p* < .001, d*f* = 76, *χ*^2^/d*f* = 9.261, CFI = .919, TLI = .903, SRMR = .047, RMSEA (90% CI) = .083 (.077, .089)—and the relative fitting indices of CFI and TLI were all above .90.

#### Criterion validity

Positive ideation was negatively related to negative suicidal ideation (− .476) and suicide probability (− .409), and positively related to and self-esteem (.567). Negative suicidal ideation was negatively related to self-esteem (− .509), and positively related to suicide probability (.707). All correlations were statistically significant (*p* < .01). The Pearson correlation matrix of variables is shown in Table 3.

### Measurement invariance

Testing was conducted based on gender, family structure (i.e., single-parent and non-single-parent households) and age.

#### Configural invariance

The baseline models of gender, single-parent/non-single-parent household and age samples were combined into multiple sets of confirmatory factor analysis models, and no restrictions were imposed on those groups of parameters. The results showed that the model fit was good (CFI_gender_ = .927, TLI_gender_ = .913, SRMR_gender_ = .051, RMSEA_gender_ = .059; CFI_family_ = .925, TLI_family_ = .910, SRMR_family_ = .051, RMSEA_family_ = .062; CFI_age_ = .919, TLI_age_ = .904, SRMR_age_ = .053, RMSEA_age_ = .061), and the fit indices are presented as Model 1, with configural invariance shown in Table 4. These results show that the PANSI-C is morphologically invariant among these groups, that is, suicidal ideation in these groups can be measured by the 14 items of the PANSI-C and the same factor structure.

**Table 4:** Measurement invariance testing results of the PANSI-C across different groups

Model	*χ*^2^	d*f*	TLI	CFI	SRMR	RMSEA	∆TLI	∆CFI	∆RMSEA
Across gender									
Model 1	823.480	152	.913	.927	.051	.059	–	–	–
Model 2	841.111	164	.918	.926	.056	.057	+ .005	− .001	− .002
Model 3	867.567	176	.919	.921	.058	.057	+ .001	− .005	0
Model 4	899.094	190	.929	.926	.058	.053	+ .01	+ .005	− .004
Across family structure									
Model 1	805.982	152	.910	.925	.051	.062	–	–	–
Model 2	828.913	164	.915	.924	.057	.060	+ .005	− .001	− .002
Model 3	844.225	176	.918	.921	.057	.059	+ .003	− .003	− .001
Model 4	887.175	190	.926	.923	.058	.056	+ .008	+ .002	− .003
Across age									
Model 1	875.323	152	.904	.919	.053	.061	–	–	–
Model 2	909.603	164	.907	.916	.061	.060	+ .003	− .003	− .001
Model 3	924.590	176	.910	.913	.062	.059	+ .003	− .003	− .001
Model 4	947.191	190	.922	.918	.062	.055	+ .012	+ .005	− .004

Model 1, configural invariance; Model 2, weak invariance; Model 3, strong invariance; Model 4, strict invariance; *χ*^2^, Chi-square goodness of fit; d*f*, degrees of freedom; TLI, Tucker–Lewis index; CFI, comparative fit index; SRMR, standardized root mean square residual; RMSEA, root mean square error of approximation; ∆TLI, TLI difference; ∆CFI, CFI difference; ∆RMSEA, RMSEA difference

#### Weak invariance

Based on Model 1, all factor loadings in each group were set to be equal, or more specifically, each item was affected by the measured latent factors in the same gender, single-parent and non-single-parent household and age groups. The model fit index is presented as Model 2 in Table 4, and the model fits well. Compared to Model 1, with ∆CFI(TLI) ≤ .01 and ∆RMSEA ≤ .015, the fit between Model 2 and Model 1 can be considered as being good, with the weak invariance of the PANSI-C between the different groups satisfied.

#### Strong invariance

Based on Model 2, all intercepts of each group of models were set to be equal. The model fit index is presented as Model 3 in Table 4, and the model fits well. Compared with Model 2, with ∆CFI(TLI) ≤ .01 and ∆RMSEA ≤ .015, it can be considered that the strong invariance is satisfied, namely the mean difference of the PANSI-C latent factors between the different groups can be expressed by the mean of the observed variables.

#### Strict invariance

In Model 3, the residual variances in the model were set to be equal. The model fit index is presented as Model 4 in Table 4, and the model fits well. Compared with Model 3, with △CFI(TLI) ≤ .01 and △RMSEA ≤ .015 for gender and different family structure, △TLI =  + .012 > .01, △CFI =  + .005 ≤ .01 and △RMSEA = − .004 ≤ .015 for age. It can be considered that gender and family structure groups meet strict invariance, namely the differences between the two groups of variation in the PANSI-C observation variable fully reflect the variation of the latent factor.

The test of measurement invariance showed that the PANSI-C met the strict invariance of gender, single-parent and non-single-parent household groups, and strong invariance of age groups.

## Discussion

The PANSI-C is a self-report instrument used to evaluate both the protective factors and negative risk factors associated with suicidal behaviors among adolescents [35]. The current study examined the psychometric properties of the PANSI-C, especially its measurement invariance, across different samples (i.e., gender, single-parent and non-single-parent households and age). This is the first study to explore the measurement invariance of the PANSI (as the PANSI-C). Our results verify the two-factor structure of the PANSI-C and support the scalar invariance of the PANSI-C in different samples. The results of the current study show that the PANSI-C has good reliability and validity in a sample of Chinese adolescents.

The results of the current study showed that, in terms of reliability, the Cronbach’s alpha coefficients for both the PANSI-NSI and PANSI-PI were acceptable, at .915 and .696, respectively, which is consistent with previous research findings [34, 35, 43]. Compared with previous studies, the reliability of the PANSI-PI subscale in this study was lower, which may be due to the fact that the participants were normal middle school students and do not understand the positive items of suicidal ideation.

In terms of validity, confirmatory factor analysis fitting indicators were good, indicating that the PANSI-C has good structural validity, which is the same as the structure obtained from the original scale and previous studies [1, 6, 30, 33–35, 43, 49]. It shows that the two-factor structures of the PANSI were also supported in Chinese adolescents. According to correlation analysis, positive ideation was negatively related to negative suicidal ideation and suicide probability, and positively related to and self-esteem. Negative suicidal ideation was negatively related to self-esteem, and positively related to suicide probability, this was consistent with previous studies [23, 35, 43]. This also verified the good construct validity of the PANSI in China.

Although some studies have compared the differences in the PANSI scores between genders, the conclusions have been inconsistent [1, 6]. The measurement invariance of a scale should be checked before any comparison of scale scores for different groups is made [27]. Therefore, we examined the measurement invariance of the PANSI-C between different samples (i.e., gender, single-parent and non-single-parent households and age). We gradually establish four models, namely configural invariance, weak invariance, strong invariance and strict invariance. The results of the configural invariance evaluation show that the number of factors and factor model were equal in gender, single-parent and non-single-parent households samples. The evaluation of weak invariance showed that the scale observation items and potential factors were equivalent in different samples. The evaluation of strong invariance showed cross-group differences in the mean of observed variables reflected the intra-group difference of the mean of potential variables. When strict invariance is obtained, the error variance of each group meets the cross-group equivalent. The results of measurement invariance show that the composition of latent variables, factor loadings, intercept and error variance of the PANSI-C in gender, single-parent and non-single-parent household samples were equal, indicating that the severity of suicidal ideation in different groups can be accurately compared when the PANSI-C is used. In addition, we obtained strong invariance across age groups, indicating that different age groups have the same reference point, so that the latent variable scores estimated by the observed variables are unbiased and the comparison between groups is meaningful. In short, the measurement invariance of the PANSI-C among different samples is obtained. The measurement results of the PANSI-C can be directly compared between different gender, family structure and age groups.

In summary, the PANSI-C consists of two dimensions, positive ideation and negative suicidal ideation, and shows good reliability and validity in the samples used in the current study. It meets the requirements of psychological measurement theory, and is a reliable and effective instrument for detecting the occurrence of suicidal ideation in Chinese adolescents, and can be applied to the psychological assessment of suicidal ideation in Chinese adolescents as well as in other related research fields. Nevertheless, several issues needed to be considered when interpreting these results. First, it lacks popularization to the other samples in China. Future research should be conducted on more representative and larger samples. Second, further studies are needed to explore the measurement invariance of PANSI-C in more groups, for providing evidence for cross-group research.

## Conclusion

In conclusion, the current study examined the psychometric properties of the PANSI-C in Chinese adolescents, looking in particular at its measurement invariance across samples determined by gender, single-parent/non-single-parent households and age. This effort broadens the psychometric and measurement properties of the PANSI-C, which could be meaningful for future empirical study into suicidal ideation prevention and treatment. Our results support the measurement invariance of the PANSI-C in the different samples, the findings indicate that the PANSI-C is a valid measure of suicidal ideation.

## Data Availability

The datasets used and/or analyzed during the current study available from the corresponding author on reasonable request.

## Abbreviations

PANSI: Positive and Negative Suicide Ideation
PANSI-C: Chinese version of the Positive and Negative Suicide Ideation Inventory
RSE: Rosenberg self-esteem scale
SPS: Suicide probability scale
WHO: World Health Organization
BSI: Beck scale for suicide ideation
SIQ: Suicidal ideation questionnaire
MSSI: Modified scale for suicide ideation
SIS: Suicidal ideation scale
PANSI-NSI: PANSI-negative suicide ideation
PANSI-PI: PANSI-positive ideation
SPSS: Statistical packages for social sciences
ML: Maximum likelihood
*χ*^2^: Chi-square goodness of fit
d*f*: Degrees of freedom
TLI: Tucker–Lewis index
CFI: Comparative fit index
SRMR: Standardized root mean square residual
RMSEA: Root mean square error of approximation
∆TLI: TLI difference
∆CFI: CFI difference
∆RMSEA: RMSEA difference
